# Sexual Satisfaction and Orgasm Experiences During Partnered and Solo Sex Among 27,931 Users of the Flo App

**DOI:** 10.1007/s10508-025-03393-y

**Published:** 2026-06-09

**Authors:** Yella Hewings-Martin, Adam C. Cunningham, Jordan Rullo, Karen L. Blair, Carley Prentice, Anna Klepchukova, Liudmila Zhaunova

**Affiliations:** 1 Flo Health UK Limited, International House, 4th Floor, 1 St. Katharine’s Way, London, E1W 1UN UK; 2 Department of Psychology, The University of Utah, Salt Lake City, UT USA; 3 Department of Psychology, Trent University, Peterborough, ON Canada

**Keywords:** Female orgasm, Orgasm frequency, Female sexual satisfaction, Masturbation, Flo App

## Abstract

Orgasm frequency during partnered sexual activities is thought to be a predictor of sexual satisfaction. Less is known about the relationship between orgasms from solo sexual activities and satisfaction. The current study examined the interplay between orgasms and sexual satisfaction in partnered and solo contexts, the role of sexual variety, and distinct sexual activities in a sample of 27,931 Flo app users (99% female, 96% women). Participants (M age, 28.14 years) reported engaging in an average of 7.79/13 partnered and 6.45/13 solo sexual activities in the past six months. Clitoral stimulation by a partner was the most frequent partnered activity (90.2%), and clitoral stimulation by hand was the most frequent solo activity (84.6%). Orgasm frequency was associated with satisfaction with a moderate effect size. Solo orgasm frequency had a weak, negative association with satisfaction. Solo sexual variety was not a significant predictor of overall satisfaction nor orgasm frequency. Partnered orgasm frequency was a significant, positive predictor of satisfaction. Greater variety in partnered sexual activities was associated with higher satisfaction and higher orgasm frequency. During solo activities, the highest orgasm frequencies were associated with clitoral stimulation using a vibrator/dildo, watching porn/erotica, and clitoral stimulation by hand. During partnered activities, using sex toys, vaginal penetration with clitoral stimulation, and masturbation with partner present had the highest orgasm frequencies. When comparing similar solo and partnered activities, orgasm frequency was consistently higher when solo. Our findings offer insights into the nuanced interplay between solo and partnered sexual experiences, the importance of orgasms, and sexual satisfaction.

**Supplementary Information:**

The online version contains supplementary material available at https://doi.org/10.1007/s10508-025-03393-y.

## Introduction

Sexual health is a fundamental human right and encompasses multiple dimensions pertaining to one’s sexuality, beyond the mere absence of disease, dysfunction, or infirmity (World Health Organization, 2015, 2017). Previous research indicates that the majority of people attribute importance to their sex lives (Wylie, 2009), and engaging in sexual activity has been linked with several potential health benefits, including lower risk for hypertension, improvements in general well-being, mental health, and relationship satisfaction, as well as modulations in immune function (Brody, 2010; Diamond & Huebner, 2012; Huebner & Rullo, 2025; Liu et al., 2016; Lorenz et al., 2018).

Sexual satisfaction is a central aspect of sexual health and is associated with overall health and quality of life (Buczak-Stec et al., 2019; Flynn et al., 2016). For example, in a study by Buczak-Stec et al. (2019), sexual satisfaction predicted participants’ self-reported satisfaction with their lives. Roughly 42–55% of women report being sexually satisfied, with the highest number of women (83%) reporting satisfaction during the first six months of a long-term relationship (Flynn et al., 2016, 2017; Frederick et al., 2017; Mulhall et al., 2008; Wylie, 2009). However, sexual problems are common, affecting around 40% of women (Nobre et al., 2006; Shifren et al., 2008), and can present a barrier to sexual satisfaction.

Existing research reports that orgasm during partnered sex is an important predictor of sexual satisfaction (Dienberg et al., 2023; Frederick et al., 2017; Kontula & Miettinen, 2016; Leonhardt et al., 2018, 2024; Macedo et al., 2023; Pascoal et al., 2018). Self-reported orgasm frequencies among women in mixed-sex relationships range between 45–71% (Blair et al., 2018; Garcia et al., 2014; Kontula & Miettinen, 2016; McElroy & Perry, 2024; Shaeer et al., 2020) and up to 86% among women in same-sex relationships (Blair et al., 2018; Frederick et al., 2018; Garcia et al., 2014). However, research robustly reports that women reach orgasm less frequently during partnered sexual activity as compared to solo sexual activity. In a recent review, McElroy and Perry highlighted that between 38 and 92% of women report usually or always experiencing orgasm during masturbation (McElroy & Perry, 2024).

If orgasm frequency during *partnered* sex serves as a predictor of sexual satisfaction, how does orgasm frequency during masturbation relate to sexual satisfaction? Lower sexual satisfaction has been linked with higher masturbation frequency (Brody, 2010; Velten & Margraf, 2017) and difficulties in reaching orgasm during masturbation (Rowland et al., 2020). In addition, Kontula and Miettinen (2016) reported that women who masturbate more frequently had fewer orgasms during partnered sex and that women who never masturbate or only did so a long time ago were more likely to reach orgasm during partnered sex (Kontula & Miettinen, 2016). A recent systematic review on masturbation among women, both partnered and single, identified that one-third of studies on this topic report a negative association between masturbation and sexual satisfaction. The remaining studies reported either no association (40%) or a positive association (26.7%). The authors purport that a negative relationship between masturbation and sexual satisfaction may be driven by the use of masturbation as a compensatory strategy (i.e., masturbation compensates for dissatisfying or unfulfilling sexual experiences or relationships) versus a complementary strategy (i.e., masturbation enhances partner sexual experiences) (Cervilla et al., 2024). Masturbation may also serve as a compensatory strategy in the absence of access to partnered sex. Overall, sexual satisfaction does not have a one-to-one relationship with orgasm frequency (regardless of orgasm context). However, Fischer and Træen (2022) point to a more nuanced, non-linear relationship between masturbation frequency and sexual satisfaction (Fischer & Træen, 2022). In their study, high masturbation frequency and high sexual satisfaction were linked with a greater variety of sexual activities, more frequent partnered sex, and being divorced or separated. High masturbation frequency and low satisfaction were linked with higher education levels, while low satisfaction, regardless of masturbation frequency, was linked with distressing sexual problems and negative body image. Thus, regarding masturbation, orgasm, and sexual satisfaction, the research findings are mixed.

To complicate this picture further, with few exceptions (e.g., Blair et al., 2018; Frederick et al., 2017; Holmberg & Blair, 2009), previous studies have been limited in the range of sexual activities assessed, focusing predominantly on partnered sexual activities (e.g., Garcia et al., 2014). How does increased orgasm frequency during other sexual activities beyond those previously studied relate to sexual satisfaction? Although there is evidence that greater sexual variety during partnered sexual activity is associated with higher orgasm frequency (Willis et al., 2018) and greater sexual satisfaction (Frederick et al., 2017; Townes et al., 2020), few research studies have specifically compared a wide range of sexual activities and their relationship to orgasm and sexual satisfaction, during both partnered and solo sex. One study (Blair et al., 2018) compared sexual activities and their relationship to orgasm and sexual satisfaction among individuals in same-sex and mixed-sex relationships. The authors found that women, regardless of relationship type (same or mixed), reported no differences in orgasm frequency or level of satisfaction with masturbation. However, differences in orgasm frequency emerged for both groups with partnered sex. Women in mixed-sex relationships reported significantly more partnered sexual activities that did not result in orgasm compared with women in same-sex relationships, but levels of sexual satisfaction did not differ between the groups.

Other studies that have focused on reporting the frequency of sexual activities have found that among cisgender heterosexual women, vaginal intercourse has been reported as the most frequent partnered sexual activity (Herbenick et al., 2010; Richters et al., 2006). Yet, vaginal penetration is not what women report as the most common sexual activity to lead to orgasm. A U.S. study with 1,055 women reported that vaginal intercourse alone was sufficient to achieve orgasm for only 18.4% of participants, whereas 36.6% said that additional clitoral stimulation during intercourse was necessary (Herbenick et al., 2018). During solo self-stimulation, which is a much more reliable source of orgasm, it is clitoral stimulation that is engaged in by most women (Burri & Carvalheira, 2019; Rowland et al., 2020). However, clitoral stimulation is not the sole factor linked with greater orgasm frequency. For example, a large U.S. study with 52,588 male and female participants found that women reported more frequent orgasms when they received oral sex more frequently, had good communication strategies with their partner, engaged in fantasy, tried anal stimulation, and when they reported high levels of relationship satisfaction (Frederick et al., 2018). Overall, the relationship between other sexual activities, orgasms, and sexual satisfaction during both partnered and solo sex deserves further attention.

### Current Study

There is a gap in research that addresses the interplay between different types of sexual activities in solo and partnered contexts and their relationship with orgasm. If orgasm frequency is a strong predictor of sexual satisfaction, then regardless of sexual experience, whether partnered or solo, and type and repertoire of sexual activity, more orgasms should equate to greater sexual satisfaction. Thus, in our study, we assessed a wide variety of solo and partnered sexual activities to answer four research questions.

RQ1) Is there an association between the overall frequency of orgasms experienced and overall sexual satisfaction ratings? Ample research points to the association of these two variables, and thus, we hypothesize that greater orgasm frequency (across contexts) will be associated with higher overall sexual satisfaction ratings (*H*1).

RQ2) Are orgasms from solo and partnered activities associated with overall sexual satisfaction, and does one have a stronger association than the other? Given that some research has indicated that increased solo sexual activity among partnered individuals can be associated with decreased sexual satisfaction (Brody & Costa, 2009; Velten & Margraf, 2017), we leave the direction of association between solo orgasm frequency and overall sexual satisfaction as an open question *(H*2). We hypothesize that greater orgasm frequency from partnered sexual activity will be associated with greater overall sexual satisfaction (*H*3) and that orgasm frequency from partnered sexual activities will have a stronger association with overall sexual satisfaction than orgasm frequency from solo activities (*H*4).

RQ3) Is engaging in a more varied repertoire of sexual activities associated with greater sexual satisfaction and orgasm frequency? We hypothesize that increased variety in solo (*H*5) and partnered (*H*6) contexts will be associated with greater overall sexual satisfaction. Additionally, we hypothesize that a variety of sexual activities in the partnered context will have a stronger association with overall sexual satisfaction (*H*7), given past research demonstrating that greater variety is associated with increased orgasm frequency and that increased orgasm frequency is generally associated with increased sexual satisfaction. Finally, we hypothesize that greater sexual variety in each context will be independently associated with greater overall orgasm frequency (*H*8; solo variety/ *H*9; partnered variety).

RQ4) Which activities are more strongly associated with the frequency of orgasms? We hypothesize that activities (either solo or partnered) that involve clitoral stimulation will have a stronger association with overall orgasm frequency than activities that only include forms of penetration without clitoral stimulation (*H*10). How the solo and partnered versions of similar activities are associated with orgasm frequency and sexual satisfaction we leave as an open research question.

## Method

The current study was conducted in partnership with the Flo smartphone app (Flo Health Ltd.), a period tracking and reproductive health app.

### Participants

Survey respondents were recruited via an in-app advertisement message with a link to a SurveyMonkey survey. Eligible participants were Flo app users over 18 who used the English language version of the app.

Between April 11, 2024, and May 5, 2024, in-app advertisement messages invited all eligible app users to participate in a study about female orgasms. Participants were then presented with a consent form that described the aims and purposes of the study and explained that their app data would be linked to their survey responses using a unique pseudonymized identifier. Specifically, user data on geographic location and age were used in the study. All other reported demographics were collected during the survey itself.

A total of 70,424 responses to the survey were collected. Responses were excluded from the analysis if they did not complete all questions in the survey, if the respondent did not have a Flo app ID, or if two attention check questions included in the survey were answered incorrectly. After exclusions, a total of 27,931 responses were included in the final analysis. Supplementary Figure A1 shows how many survey responses were excluded and the reasons for exclusion.

Table 1 shows a breakdown of participants’ ages, ethnicity, education level, biological sex, gender, sexuality, and sexual partner’s biological sex. Most participants (38%) were between 18 and 25 years old, with a mean age of 28.14 (*SD* = 6.80). The majority identified as White, European American, or Caucasian (56%), and held a bachelor’s degree (35%) as their highest education level attained.

**Table 1:** Demographic summary of respondents, including age, ethnicity, education level, biological sex, gender, sexuality, and partner sex

	Total N = 27,931	
	n	**%**
*Age bands* (in years)		
18–25	10,612	38.0
26–30	7426	26.6
31–35	5552	19.9
36–40	2435	8.7
41–45	971	3.5
46 +	378	1.4
Missing	557	2.0
*Ethnicity*		
American Indian or Alaskan Native	152	0.5
Another Indigenous background	189	0.7
Asian (including East Asian, South Asian and Southeast Asian) or Asian American	2507	9.0
Black or African or African American	3852	13.8
Hispanic, Latino, or Spanish origin	1646	5.9
I prefer not to say	535	1.9
Middle Eastern/North African or Middle Eastern/North African American	445	1.6
Multi- or Bi-racial	2114	7.6
Native Hawaiian or Other Pacific Islander	65	0.2
Other	879	3.1
White, European American, or Caucasian	15,547	55.7
*What is the highest level of education you’ve completed?*		
Associate’s degree	2117	7.6
Bachelor’s degree	9741	34.9
Doctorate degree	611	2.2
High school graduate, diploma, or the equivalent (e.g., GED)	8523	30.5
I prefer not to say	648	2.3
Master’s degree	4307	15.4
Other (please specify)	640	2.3
Some high school, no diploma	1344	4.8
*What is your biological sex?*		
Female	27,617	98.9
Intersex	10	0.0
Male	227	0.8
Other (please specify)	37	0.1
Prefer not to answer	40	0.1
*Gender*		
Agender	19	0.1
Gender Queer or Gender Fluid)	94	0.3
Man	145	0.5
Multiple Responses	490	1.8
Non-Binary	173	0.6
Other	17	0.1
Prefer not to answer	30	0.1
Unsure/Questioning	36	0.1
Woman	26,927	96.4
*Sexuality*		
Asexual	131	0.5
Bisexual/Pansexual	6455	23.1
Gay or lesbian	314	1.1
Heterosexual	19,756	70.7
Other	1144	4.1
Prefer not to answer	131	0.5
*What is your partner’s biological sex?*		
Female	433	1.6
Intersex	5	0.0
Male	22,703	81.3
Other (please specify)	28	0.1
Prefer not to answer	29	0.1
Missing	4733	16.9
*In the past 6 months, about how many times have you engaged in any type of sexual activity? (With a partner)*		
About once a month	2257	8.1
About once a week	5254	18.8
Four or more times a week	2678	9.6
Never	2306	8.3
Once or twice	2284	8.2
Prefer not to answer	201	0.7
Two or three times a month	5600	20.0
Two or three times a week	7119	25.5
Missing	232	0.8
*In the past 6 months, about how many times have you engaged in any type of sexual activity? (On your own (solo))*		
About once a month	3750	13.4
About once a week	4647	16.6
Four or more times a week	2159	7.7
Never	3154	11.3
Once or twice	3758	13.5
Prefer not to answer	303	1.1
Two or three times a month	5297	19.0
Two or three times a week	4631	16.6
Missing	232	0.8
*Over the past 6 months, how satisfied have you been with your overall sexual life?*		
1 = Very dissatisfied	1891	6.8
2 = Moderately dissatisfied	3552	12.7
3 = About equally satisfied and dissatisfied	4968	17.8
4 = Moderately satisfied	8470	30.3
5 = Very satisfied	7224	25.9
Prefer not to answer	451	1.6
Missing	1375	4.9
*Over the past 6 months, when you had sexual stimulation or intercourse, how often did you reach orgasm (climax)?*		
0 = No sexual activity	308	1.1
1 = Almost never or never	3311	11.9
2 = A few times (less than half the time)	2430	8.7
3 = Sometimes (about half the time)	3634	13.0
4 = Most times (more than half the time)	6389	22.9
5 = Almost always or always	10,306	36.9
Prefer not to answer	178	0.6
Missing	1375	4.9

### Measures

The following measures were collected and analyzed in the study. All survey questions included a “prefer not to answer” response option, which was not assigned a numerical value. When participants selected this response option, their response was not included in the calculated mean score for that particular scale or question.

### Solo and Partnered Sexual Activities

Participants were asked to report on the frequency of engaging in any type of sexual activity in both partnered or solo contexts. Answer options were: never, once or twice, about once a month, two or three times a month, about once a week, two or three times a week, four or more times a week. To assess the sexual activities engaged in by participants over the past 6 months, participants completed a measure created for the current study through internal study team discussions assessing the frequency with which they engaged in 13 partnered and 13 solo sexual behaviors. They were asked “When you’re engaging in sexual activity with a partner, how often do you do the following?” for partnered activities and presented with the following options: vaginal penetration (with a penis, fingers, sex toy, etc.) without stimulation of any other body part; vaginal penetration (with a penis, fingers, sex toy, etc.) with clitoral stimulation; vaginal penetration (with a penis, fingers, sex toy, etc.) with stimulation of another body part (e.g., breast, nipples, ears, neck, anal, etc.); G-spot stimulation; anal penetration/stimulation; clitoral stimulation/clitoral strokes by a partner’s mouth/tongue/hand; giving oral sex to a partner; giving oral sex to a partner while receiving oral sex; masturbation (clitoral or vaginal) with a partner present; Simultaneous use of a sex toy while engaging in any of the activities above; Porn/Erotica (visual, audio, or literature) while engaging in any of the activities above; Fantasy, role-play, or other imaginative play while engaging in any of the activities above; Kinks/fetish sex while engaging in any of activities above. For solo sexual activities, they were asked “When you’re engaging in sexual activities on your own (solo), how often do you do the following?” and presented the following options: clitoral stimulation by hand; clitoral stimulation with the use of a vibrator/dildo; clitoral stimulation with the use of another object or by other means (pillow, shower faucet, rubbing thighs together, during exercise etc.); vaginal penetration (with a sex toy, fingers, etc.) without stimulation of any other body part; vaginal penetration (with a sex toy, fingers, etc.) with clitoral stimulation; vaginal penetration (with a sex toy, fingers, etc.) with stimulation of another body part (e.g., breast, nipples, ears, neck, anal, etc.) and/or with simultaneous use of a sex toy; G-spot stimulation; anal penetration/stimulation, Watching porn/erotica while engaging in any of the activities above; Listening to porn/erotica while engaging in any of the activities above; Reading porn/erotica while engaging in any of the activities above; Fantasy, role-play, or other imaginative play while engaging in any of the activities above; Kinks/fetish sex while engaging in any of activities above. Answer options with their assigned numerical values were as follows: 0 = never, 1 = I have done this but not in the past 6 months, 2 = sometimes, 3 = often, 4 = every time. Table 2 lists each of the activities assessed and the breakdown of responses. For analysis purposes, we explore overall engagement frequency, defined as the proportion of participants who engage in an activity at least “sometimes,” and consistent engagement frequency, defined as the proportion of participants who reported engaging in an activity “every time” they engaged in sexual activities (e.g., every time they had partnered sex or every time they engaged in solo sexual activity). In addition, mean frequency scores were calculated which represents the numerical mean of the numerically coded responses.

**Table 2:** Frequency of engaging in each solo and partnered activity

Category	Activity	n/%	Never	I have done this, but not in the past 6 months	Sometimes	Often	Every time	Prefer not to answer	Missing	Mean frequency score	SD frequency score
Partnered	Anal penetration or stimulation	n	13,742	2985	5802	886	683	135	3698	0.82	1.08
		%	56.71%	12.32%	23.94%	3.66%	2.82%	0.56%	NA		
	Clitoral stimulation by partner	n	1089	1198	6465	8379	7020	82	3698	2.78	1.05
		%	4.49%	4.94%	26.68%	34.58%	28.97%	0.34%	NA		
	Fantasy or role-play	n	17,131	2078	4079	684	162	99	3698	0.53	0.91
		%	70.69%	8.58%	16.83%	2.82%	0.67%	0.41%	NA		
	G-spot stimulation	n	6488	1175	8146	5080	2809	535	3698	1.85	1.34
		%	26.77%	4.85%	33.62%	20.96%	11.59%	2.21%	NA		
	Giving oral Sex	n	1686	1508	8265	8815	3860	99	3698	2.48	1.05
		%	6.96%	6.22%	34.11%	36.38%	15.93%	0.41%	NA		
	Giving oral sex while receiving oral sex	n	7497	3975	9875	2342	423	121	3698	1.34	1.07
		%	30.94%	16.4%	40.75%	9.66%	1.75%	0.5%	NA		
	Kinks or fetish activities	n	14,401	1913	5691	1585	477	166	3698	0.82	1.11
		%	59.43%	7.89%	23.48%	6.54%	1.97%	0.69%	NA		
	Masturbation with partner present	n	8996	2328	9080	2861	865	103	3698	1.34	1.19
		%	37.12%	9.61%	37.47%	11.81%	3.57%	0.43%	NA		
	Porn or Erotica with partner	n	15,590	2645	4791	928	163	116	3698	0.64	0.96
		%	64.33%	10.91%	19.77%	3.83%	0.67%	0.48%	NA		
	Using sex toys during activities	n	10,991	2612	6692	2952	886	100	3698	1.17	1.23
		%	45.36%	10.78%	27.62%	12.18%	3.66%	0.41%	NA		
	Vaginal penetration only	n	4432	1006	4611	4449	9628	107	3698	2.57	1.49
		%	18.29%	4.15%	19.03%	18.36%	39.73%	0.44%	NA		
	Vaginal penetration and clitoral stimulation	n	1456	856	5429	7201	9192	99	3698	2.90	1.13
		%	6.01%	3.53%	22.4%	29.72%	37.93%	0.41%	NA		
	Vaginal penetration and other body part stimulation	n	1410	897	5472	7339	9022	93	3698	2.89	1.12
		%	5.82%	3.7%	22.58%	30.29%	37.23%	0.38%	NA		
Solo	Anal penetration or stimulation	n	20,587	1482	3069	430	139	216	2008	0.36	0.79
		%	79.42%	5.72%	11.84%	1.66%	0.54%	0.83%	NA		
	Clitoral stimulation by hand	n	2368	1512	6337	4069	11,525	112	2008	2.80	1.31
		%	9.13%	5.83%	24.45%	15.7%	44.46%	0.43%	NA		
	Clitoral stimulation by other means	n	9830	3373	8169	2739	1672	140	2008	1.34	1.26
		%	37.92%	13.01%	31.51%	10.57%	6.45%	0.54%	NA		
	Clitoral vibrator/dildo	n	7374	1668	4514	5137	7106	124	2008	2.11	1.58
		%	28.45%	6.43%	17.41%	19.82%	27.41%	0.48%	NA		
	Fantasy, role-play, imaginative play	n	16,183	915	5177	2249	1147	252	2008	0.88	1.25
		%	62.43%	3.53%	19.97%	8.68%	4.42%	0.97%	NA		
	G-spot stimulation	n	13,526	1477	6570	2332	1488	530	2008	1.08	1.29
		%	52.18%	5.7%	25.34%	9%	5.74%	2.04%	NA		
	Kinks or fetish	n	19,517	738	3646	1162	519	341	2008	0.53	1.02
		%	75.29%	2.85%	14.06%	4.48%	2%	1.32%	NA		
	Listening to Porn/Erotica	n	11,831	1714	6382	3472	2315	209	2008	1.32	1.39
		%	45.64%	6.61%	24.62%	13.39%	8.93%	0.81%	NA		
	Reading Porn/Erotica	n	12,389	2442	6929	2912	1052	199	2008	1.13	1.24
		%	47.79%	9.42%	26.73%	11.23%	4.06%	0.77%	NA		
	Vaginal penetration only	n	12,216	1960	7649	2521	1424	153	2008	1.18	1.27
		%	47.12%	7.56%	29.51%	9.72%	5.49%	0.59%	NA		
	Vaginal penetration and clitoral stimulation	n	6976	1994	8152	4740	3901	160	2008	1.86	1.39
		%	26.91%	7.69%	31.45%	18.28%	15.05%	0.62%	NA		
	Vaginal penetration with stimulation of another body part	n	11,376	1703	7992	3069	1587	196	2008	1.29	1.30
		%	43.88%	6.57%	30.83%	11.84%	6.12%	0.76%	NA		
	Watching Porn/Erotica	n	5268	1825	7191	6514	4931	194	2008	2.15	1.37
		%	20.32%	7.04%	27.74%	25.13%	19.02%	0.75%	NA		

SD = Standard Deviation. Possible range of the mean frequency score = 0–4

### Orgasms and Sexual Satisfaction

Participants were asked questions about their overall satisfaction with their sex life and the frequency of reaching orgasm during sexual activity over the previous 6 months. Answer options were assigned a numerical value as presented below. Numerical values were used to calculate mean scores.

*Sexual Satisfaction*. To assess sexual satisfaction, participants were asked the following question: “Over the past 6 months, how satisfied have you been with your overall sexual life?” Answer options: 5 = Very satisfied, 4 = Moderately satisfied, 3 = About equally satisfied and dissatisfied, 2 = Moderately dissatisfied, 1 = Very dissatisfied.

*Orgasm Frequency*. To assess orgasm frequency, participants were asked the following question: “Over the past 6 months, when you had sexual stimulation or intercourse, how **often** did you reach orgasm (climax)?” Answer options: 0 = No sexual activity, 5 = Almost always or always, 4 = Most times (more than half the time), 3 = Sometimes (about half the time), 2 = A few times (less than half the time), 1 = Almost never or never. In addition, participants were asked to indicate how frequently they reached orgasms from each of the sexual activities that they said they engaged in during partnered and/or solo sexual activity. The answer options and assigned numerical values were the same as for overall orgasm frequency. These values were used to construct the weighted orgasm scores (see below). All participants were also asked if, on average, they reached orgasm more frequently alone, with a partner, or about the same. Those who indicated reaching orgasm more frequently with a partner were presented with the following question: “What do you think are the main reasons that you reach orgasm more frequently with a partner: (select the top 3)” Answer options were curated by the research team and included: I don’t engage in solo sexual activities, Emotional connection/intimacy with partner, Expressing love for your partner, Partner stimulates me in a way that is adequate to reach an orgasm (including body part, foreplay, length of time, etc.), Element of surprise (increased pleasure when input isn’t something you’re controlling), Attraction to partner increases arousal and pleasure, Stimulating my partner gives me pleasure, Real-time visual and/or tactile stimulation, Other (please specify), Prefer not to answer. Those indicating that they reached orgasm more frequently alone were asked the following: “What do you think are the main reasons that you reach orgasm more frequently when you are alone: (select the top 3)” Answer options were: Partner doesn’t stimulate me in a way that is adequate to reach an orgasm (including body part, foreplay, length of time, etc.), Vaginal penetration is uncomfortable/painful, Partner reaches orgasm too soon, Partner has frequent erectile problems, I often feel preoccupied with life stressors, Problems in your relationship, Hard to communicate sexual preferences with partner, Vaginal dryness, No pressure to reach orgasm or sexually perform, Less self-conscious about body image, Feel safer to let go of control (can anticipate what will happen), Past sexual, emotional or physical abuse, Other (please specify), Prefer not to answer.

### Weighted Orgasm Scores

Using the responses outlined above, we calculated a weighted orgasm frequency score for each participant. For each individual, the numerical value of the frequency of engaging in a specific sexual activity was multiplied by the frequency of reaching orgasm during this activity. The sum of these values across the partnered and solo activities for each participant was then calculated, yielding a single orgasm frequency score (weighted by engaging in the activity) for partnered and solo activities, respectively.

### Analytic Plan

All analyses were conducted in R v. 4.4.0 (R Core Team, 2024), on a Mac OS X 14.6.1. To provide context for the reader concerning our participants’ general frequency of sexual activities across solo and partnered contexts, we use descriptive statistics to describe the range of responses provided and to compare between solo and partnered contexts. We also report participants’ perceptions of orgasm frequency in various contexts before moving on to explore our research questions and hypotheses.

Research Question 1 (the association between overall orgasm frequency and sexual satisfaction) was assessed with a Pearson’s correlation. RQs 2 and 3 were assessed with multiple regression. Table A2 in the Supplementary materials presents the correlations between all predictor and outcome variables used across both regression analyses. For RQ4, after calculating the weighted Solo and Partnered Scores, we assessed the distributions using histograms to check normality. As the distributions of Partnered Score and Solo Score deviated significantly from normal, Wilcoxon signed-rank tests were used to compare these scores and paired-samples t-tests were used to compare orgasm frequency between partnered and solo versions of similar sexual activities.

## Results

### Initial Descriptive Analyses

Respondents reported how frequently they had engaged in partnered and solo sexual activities in the past six months. The most common response for partnered sexual activity was two or three times a week (25%), while for solo sexual activity it was two to three times a month (19%). The full range of responses are shown in Table 1.

Respondents reported how frequently they engaged in a range of partnered sexual activities. Overall, clitoral stimulation by a partner was the most frequent partnered behavior, with 90.2% of respondents reporting this activity at least sometimes they engaged in partnered activity, followed by vaginal penetration with stimulation of another body part (90.1%), and vaginal penetration with clitoral stimulation (90%). Fantasy or role-play activities were the least frequently reported activities, with only 20.3% of respondents reporting this activity at least sometimes they engaged in partnered activity, and 70.7% of respondents reporting never engaging in fantasy or role-play activities. In contrast, vaginal penetration only was the activity most consistently engaged in every time participants had partnered sex (39.7%), followed by vaginal penetration with clitoral stimulation (37.9%), and vaginal penetration with stimulation of another body part (37.2%). The sexual activities with the highest mean frequency scores were vaginal penetration with clitoral stimulation (M 2.90, SD 1.13), vaginal penetration with stimulation of another body part (M 2.89, SD 1.12), and clitoral stimulation by a partner (M 2.78, SD 1.05). The full range of responses and activities are shown in Table 2.

Respondents also reported about activities they engaged in on their own. Clitoral stimulation by hand was the most frequently engaged in solo activity overall, with 84.6% of respondents reporting engaging in this activity at least sometimes. In contrast, anal penetration or stimulation was the least frequently engaged in solo activity overall reported by 14% as sometimes, often, or every time and by 79.4% as never. Notably, while vaginal penetration only was the partnered activity most consistently engaged in every time, very few participants (5.5%) reported engaging in it every time during solo sexual activity. In contrast, clitoral stimulation by hand was the most consistently engaged in activity every time (44.5%) and also had the highest mean frequency score (M 2.80, SD 1.31). The full range of responses and activities are in Table 2.

To assess sexual variety in solo and partnered sexual activities, the number of different sexual activities participants engaged in was examined separately for solo and partnered contexts. On average, participants reported engaging in 7.79 (*SD* = 2.48) of the 13 listed partnered sexual activities (range 1–13). Engagement in solo sexual activities was slightly lower, with participants reporting an average of 6.45 (*SD* = 2.78; *t*(48,509) = 56.15, *p* < .001) of the 13 listed solo activities (range: 1–13). Responses to items about partnered activities were more likely to be missing (4,416) than those about solo activities (2,813), likely reflecting differences in relationship status and access to sexual partners over the past six months. In total, 4.2% (1,173/27,931) of participants only reported engaging in partnered activities, while 9.3% (2,598/27,931) of participants reported only engaging in solo activities over the previous 6-month period.

### Sexual Satisfaction

Overall, the sample was fairly sexually satisfied (*M* = 3.60, *SD* = 1.23), with 56% of the full sample (N = 27,931) indicating that they were moderately or very satisfied with their overall sex life for the past six months. Only 7% of the sample indicated that they were very dissatisfied with their sex life during the same period. Table 1 displays the complete breakdown of responses by response option and Table A1 in the Supplementary Materials the mean sexual satisfaction score.

### Orgasm Frequency

When exploring overall orgasm frequency, 37% of the participants indicated that they almost always or always reached orgasm during their sexual activities of the past 6 months. Conversely, 21% of participants indicated that they reached orgasm less than half of the time, almost never, or never during the same period. Table 1 displays the complete breakdown of responses by response option. The mean orgasm frequency score for the past six months was 3.65 (*SD* = 1.45), indicating an average frequency well above the midpoint of the 0–5 scale, with higher scores indicating greater orgasm frequency (Table A1 in the Supplementary Materials).

While the analyses described below statistically explore the rates of orgasm frequency reported by participants for solo vs. partnered activities, we also directly asked participants whether they feel that they reach orgasm more frequently alone, with a partner, or about the same in each context. Just under half of the sample (47%) reported reaching orgasm more frequently during solo sexual activities, while only 21% reported reaching orgasm more frequently with a partner. Just under one-third of the participants (27%) reported no difference in their orgasm frequency between solo and partnered activities. The same pattern bore out when examining participants’ weighted orgasm frequency scores across solo and partnered domains of sexual activity, such that those who reported that they orgasmed more frequently with a partner also had a higher weighted orgasm frequency score than those who reported orgasming more frequently alone (partnered *M* = 49.3, solo *M* = 28.9, *t*(11,621) = 27.92, *p* < .001). The same was true for those who reported orgasming more frequently from solo activities (partnered *M* = 31.6, solo *M* = 57.5, *t*(25,902) = 84.4,* p* < .001).

### Perceived Reasons for Contextual Orgasm Frequency Differences

**Fig. 1:**
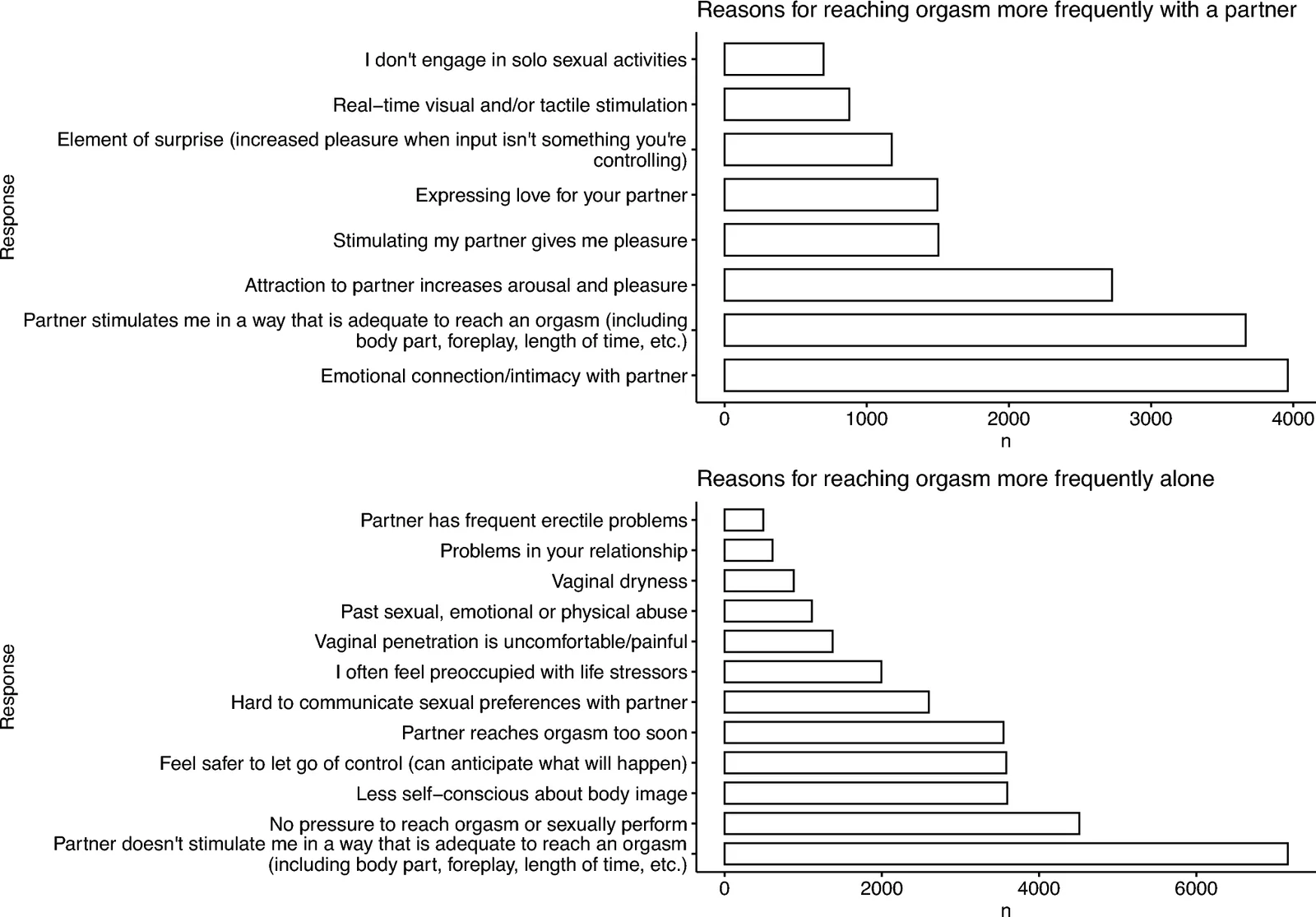
Reasons for reaching orgasm more frequently with a partner or alone

To further explore why participants reported reaching orgasm more frequently in solo versus partnered sexual contexts, those who selected either option were asked to identify up to three primary reasons for their response (from a list of 22 options). The frequency of selected reasons is visualized in Fig. 1. Among participants who reported reaching orgasm more frequently with a partner (21%), the most commonly selected reasons included: emotional connection and intimacy with their partner, partner stimulation being adequate to induce orgasm, and attraction to their partner increasing their experiences of arousal and pleasure. Among participants who reported reaching orgasm more frequently in solo sexual contexts (47%), the most commonly endorsed reasons included: partner stimulation not being adequate to induce orgasm, no pressure to reach orgasm or sexually perform, less self-consciousness about body image, and feeling safer to let go of control by having the ability to anticipate stimulation. Additionally, barriers to orgasm in partnered sex, such as partner reaching orgasm too quickly, difficulty communicating sexual preferences, preoccupation with life stressors, and vaginal penetration being uncomfortable or painful, were also frequently cited as reasons for higher orgasm frequency during solo sexual activity.

### Predictors of Overall Sexual Satisfaction

**Fig. 2:**
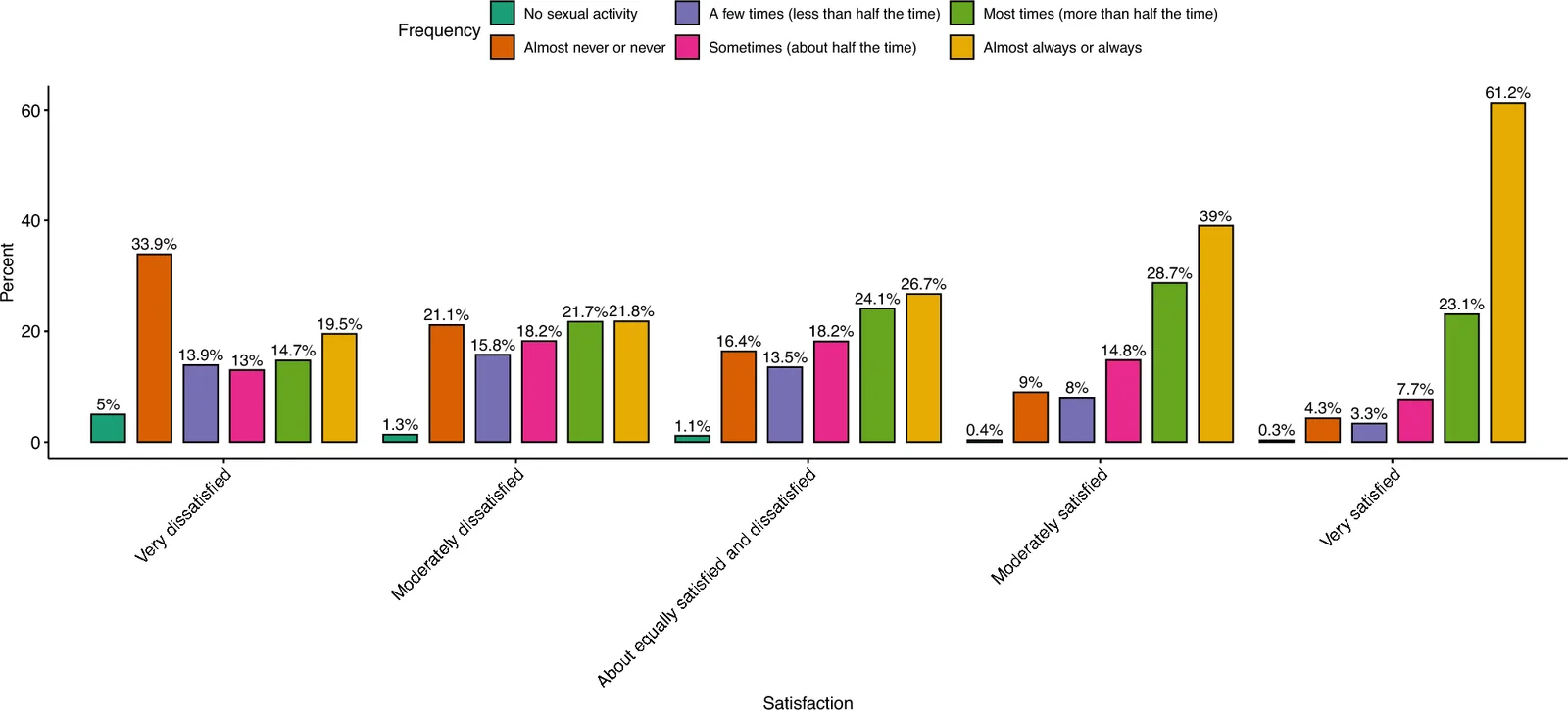
Orgasm frequency in the last 6 months plotted by satisfaction with overall sex life in the last 6 months

To assess the extent to which our sample aligned with past research, we hypothesized that overall orgasm frequency would be positively associated with overall sexual satisfaction. Using a pearson correlation, this hypothesis (*H*1) was supported (*r* = 0.38, *p* < .001, *N* = 26,007), with a moderate effect size. Figure 2 illustrates the association across response categories (e.g., “almost always or always” reaching orgasm vs. “very satisfied” with overall sex life). While the overall association is positive, the figure also highlights individual variability, showing that some individuals report high sexual satisfaction despite lower orgasm frequency and vice versa.

### Orgasm Frequency and Sexual Variety in Partnered and Solo Contexts

To assess RQ2 (*H*2-*H*4) and RQ3 (*H*5-*H*7), a hierarchical multiple regression analysis was conducted predicting sexual satisfaction from orgasm frequency and sexual variety by context, as well as their interactions (Table 3). For this analysis, the sample was limited to individuals who had engaged in partnered sexual activity in the past six months to avoid artificially low scores for partnered orgasm frequency and sexual variety (n = 19,469). This analysis does not include individuals who engaged only in solo sexual activity.

**Table 3:** Hierarchical multiple regression analysis predicting sexual satisfaction from orgasm frequency and sexual variety

	Model 1					Model 2					Model 3				
*Variable*	*b*	*SE*	*CI*	*ß*	*p*	*b*	*SE*	*CI*	*ß*	*p*	*b*	*SE*	*CI*	*ß*	*p*
(Intercept)	2.68	0.03	2.62–2.75	0.00	< 0.001	2.11	0.04	2.03–2.20	0.00	< 0.001	1.90	0.10	1.70–2.10	0.00	< 0.001
Solo score	− 0.01	0.00	− 0.02–− 0.01	− 0.03	< 0.001	− 0.01	0.00	− 0.02–− 0.00	− 0.02	0.002	0.01	0.01	− 0.01–0.02	− 0.02	0.399
Partnered score	0.15	0.00	0.14–0.15	0.34	< 0.001	0.13	0.00	0.12–0.13	0.29	< 0.001	0.15	0.01	0.13–0.17	0.30	< 0.001
Solo count						− 0.04	0.00	− 0.04–− 0.03	− 0.09	< 0.001	− 0.03	0.01	− 0.05–− 0.01	− 0.08	0.007
Partnered count						0.12	0.00	0.11–0.12	0.23	< 0.001	0.12	0.01	0.11–0.14	0.23	< 0.001
Solo score × Partnered score											− 0.00	0.00	− 0.00–− 0.00	− 0.01	0.027
Solo count × Partnered count											− 0.00	0.00	− 0.00–0.00	− 0.01	0.318
Observations	19,469					19,469					19,469				
R^2^/R^2^ adjusted	0.112/0.112					0.156/0.156					0.157/0.156				
Change in R^2^/R^2^ adjusted						0.044/ 0.044					0.001/0.000				
*F*	1231 (2, 19,466)					902 (4, 19,464)					602.9 (6, 19,462)				
AIC	59,693.379					58,702.957					58,700.957				

Model 1, which included the weighted orgasm frequency scores for solo and partnered contexts, significantly predicted sexual satisfaction, *R*^*2*^ = .112, *F*(2, 19,466) = 1231, *p* < .001. Both variables significantly predicted overall sexual satisfaction; however, their associations differed in direction. Partnered orgasm frequency was positively associated with sexual satisfaction, while solo orgasm frequency was negatively associated with sexual satisfaction.

Adding the variety variables in Model 2 resulted in a statistically significant improvement in model fit, *ΔR*^*2*^ = .044, *F*(4, 19,464) = 902, *p* < .001, bringing the total explained variance to 15.6% (*R*^*2*^ = .156). Variety in partnered sexual activities was positively associated with sexual satisfaction, while variety in solo sexual activities was negatively associated with sexual satisfaction.

Finally, the inclusion of interaction terms in Model 3 led to a small but statistically significant increase in *R*^*2*^ (*ΔR*^*2*^ = .001, *F*(6, 19,462) = 602.9, *p* < .001) for a final model *R*^*2*^ = .157. The interaction between the orgasm frequency scores was significant, while the interaction between variety scores was not. With respect to our hypotheses, *H*2 was not supported, with solo orgasm frequency not significantly predicting sexual satisfaction. *H*3 was supported, such that partnered orgasm frequency was a significant and positive predictor of overall sexual satisfaction. We also found support for *H*4, in that partnered orgasm frequency was a stronger predictor of overall sexual satisfaction than solo orgasm frequency (*Z* = 34.20, *p* < .001, *d* = 0.49). A similar pattern emerged for *H*5 and *H*6 concerning the associations between sexual variety and overall sexual satisfaction. *H*5 was not supported, as solo sexual variety was a significant negative predictor of overall sexual satisfaction. *H*6 was supported, suggesting that greater variety in partnered sexual activities is associated with higher overall sexual satisfaction. Finally, *H*7 was supported in that partnered sexual variety is a stronger predictor of overall sexual satisfaction than solo sexual variety.

### Predictors of Overall Orgasm Frequency

A multiple regression analysis was conducted to predict overall orgasm frequency from sexual variety in each context, as well as their interaction, while also controlling for relationship status (Table 4). Once again, the sample used for the regression analysis only included participants who reported some form of partnered sexual activity in the past six months (n = 20,805). The variety variables in each context reflect the total count of sexual activities that a participant had engaged in within the past six months, with a maximum of 13 possible activities in each context (solo and partnered).

**Table 4:** Multiple regression analysis predicting overall orgasm frequency from sexual variety

	Orgasm frequency				
*Predictors*	*ß*	*b*	*SE*	*CI*	*p*
(Intercept)	− 0.30	3.26	0.08	3.10–3.42	< 0.001
Solo activity count	− 0.01	− 0.01	0.01	− 0.03–0.01	0.479
Partnerned activity count	0.24	0.13	0.01	0.11–0.15	< 0.001
Relationship status [Yes]	0.34	0.48	0.03	0.42–0.53	< 0.001
Solo activity count × Partnered activity count	0.00	0.00	0.00	− 0.00–0.00	0.728
Observations		20,805			
R^2^/R^2^ adjusted		0.072/0.072			
*F*		405.4 (4, 20,800)			
AIC		71,263.770			

The overall model was statistically significant, *R*^*2*^ = .072, *F*(4, 20,800) = 405.4, *p* < .001, indicating that approximately 7.2% of the variance in overall orgasm frequency was explained by the predictors. The control variable of relationship status was a significant predictor, with those in relationships reporting higher orgasm frequency. Variety of sexual activities in the solo context was not a significant predictor of orgasm frequency, but variety of partnered sexual activities was a significant and positive predictor of overall orgasm frequency, suggesting that greater variety in partnered sexual activities is associated with more frequent orgasms. The interaction between solo and partnered variety was not significant. Consequently, *H*8 was not supported, given that there was no association between solo variety and orgasm frequency, while *H*9 was supported, with a positive association between partnered variety and orgasm frequency.

### Comparing Solo and Partnered Activities

Our final research question (RQ4) sought to better understand which specific sexual activities were most strongly associated with frequent orgasms in both solo and partnered contexts. Additionally, we hypothesized (*H*10) that activities involving clitoral stimulation would be more strongly associated with orgasm frequency than activities focusing only on penetration. Finally, we also sought to compare individual orgasm frequency scores for specific activities across their solo and partnered counterparts.

**Fig. 3:**
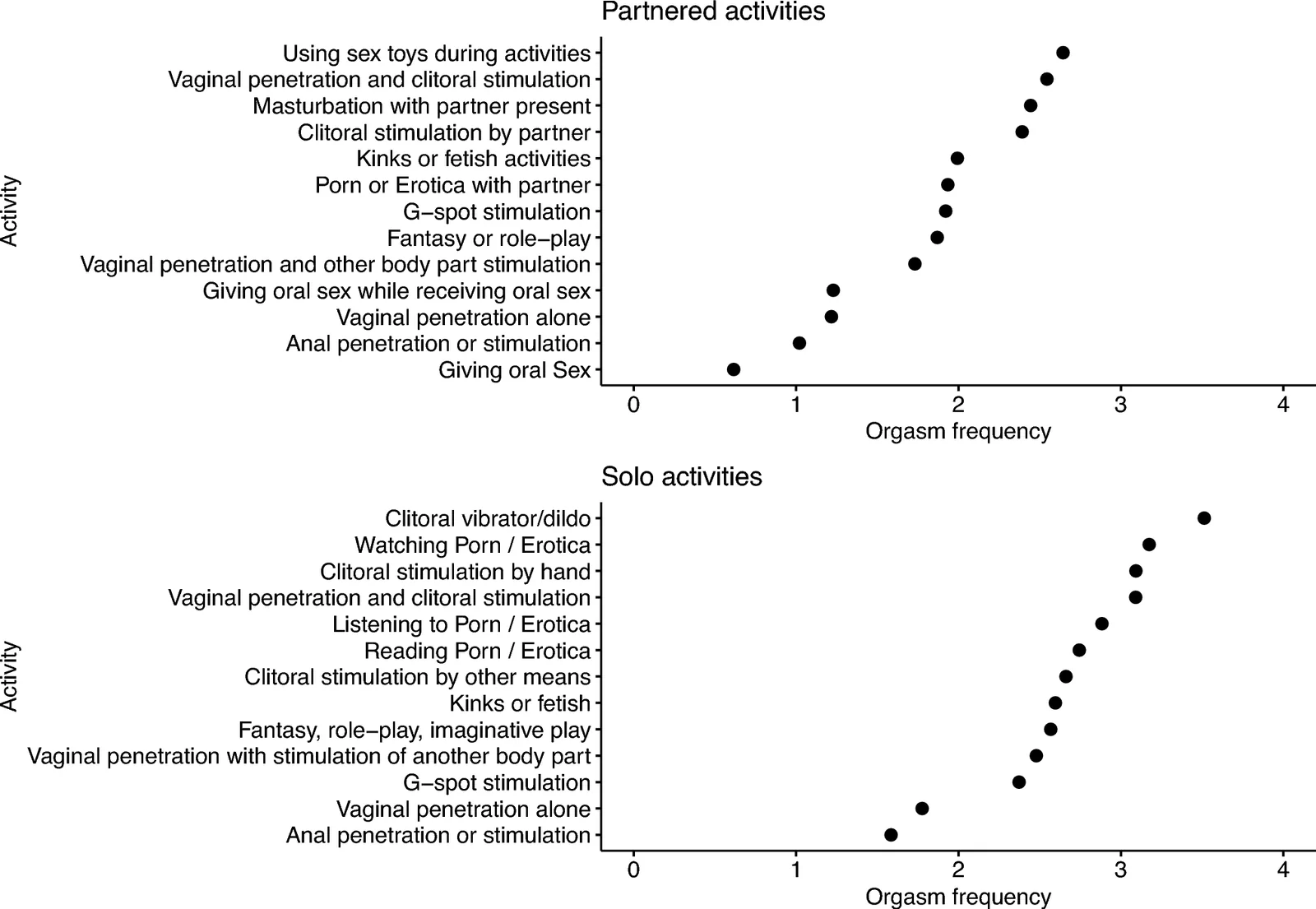
Mean Orgasm Frequencies from solo versus partnered sexual activities

To examine which sexual activities were most frequently associated with orgasm, mean orgasm frequencies were calculated separately for solo and partnered activities. The results are illustrated in Fig. 3, showing that orgasm frequency varied by activity type, with some solo activities yielding higher orgasm frequencies than their partnered counterparts. Supporting *H*10, the highest mean orgasm frequencies for solo activities were observed for clitoral stimulation using a vibrator/dildo, watching porn/erotica, and clitoral stimulation by hand. For partnered activities, the highest orgasm frequencies were reported for using sex toys during partnered sex, vaginal penetration with clitoral stimulation, and masturbation with partner present. Activities such as giving oral sex and vaginal penetration without additional stimulation had the lowest mean orgasm frequencies in both solo and partnered contexts.

To directly compare orgasm frequency between similar solo and partnered activities, a series of paired-samples *t*-tests were conducted (see Table 5). Across all comparisons, orgasm frequency was significantly higher in solo versions of the activities than in partnered versions (all *p* < .001), with effect sizes ranging from small (*d* = .16) to moderate (*d* = .73). Even for activities that can be performed in both solo and partnered context (e.g., clitoral stimulation, vaginal penetration with additional stimulation, fantasy-based activities), individuals report reaching orgasm more frequently when engaging in these activities alone than when engaging in them with a partner. This pattern is particularly pronounced for activities that involve external stimuli (e.g., vibrators, porn consumption), but holds for other activities, such as clitoral stimulation by hand, vaginal penetration, G-spot stimulation, and fantasy-kink-based activities.

**Table 5:** Paired *t* tests for orgasm frequency by similar activities (partnered vs. solo)

Partnered activity	Solo activity	n	Partnered M (SD)	Solo M (SD)	*t*	Cohen’s *d*	*p*
Clitoral stimulation by partner	Clitoral stimulation by hand	19,782	2.45 (1.55)	3.12 (1.37)	− 53.82	− 0.38	< .001
Clitoral stimulation by partner	Clitoral vibrator/dildo	16,330	2.43 (1.55)	3.54 (1.05)	− 84.99	− 0.66	< .001
Clitoral stimulation by partner	Clitoral stimulation by other means	13,281	2.40 (1.56)	2.70 (1.49)	− 18.12	− 0.15	< .001
Vaginal penetration only	Vaginal penetration only	10,217	1.41 (1.51)	1.83 (1.61)	− 24.73	− 0.24	< .001
Vaginal penetration and clitoral stimulation	Vaginal penetration and clitoral stimulation	16,186	2.66 (1.45)	3.15 (1.33)	− 39.08	− 0.30	< .001
Vaginal penetration and other body part stimulation	Vaginal penetration with stimulation of another body part	12,392	1.91 (1.58)	2.53 (1.55)	− 38.41	− 0.34	< .001
G− spot stimulation	G− spot stimulation	9182	2.18 (1.53)	2.42 (1.50)	− 15.80	− 0.16	< .001
Anal penetration or stimulation	Anal penetration or stimulation	3412	1.44 (1.51)	1.74 (1.61)	− 11.88	− 0.20	< .001
Porn or Erotica with partner	Watching Porn/Erotica	7533	2.01 (1.59)	3.23 (1.20)	− 63.10	− 0.72	< .001
Porn or Erotica with partner	Listening to Porn/Erotica	5423	2.04 (1.57)	2.91 (1.39)	− 35.78	− 0.48	< .001
Porn or Erotica with partner	Reading Porn/Erotica	4823	2.04 (1.58)	2.80 (1.41)	− 29.28	− 0.42	< .001
Fantasy or role− play	Fantasy, role− play, imaginative play	3592	2.08 (1.49)	2.65 (1.42)	− 20.35	− 0.34	< .001
Kinks or fetish activities	Kinks or fetish	4043	2.34 (1.46)	2.68 (1.39)	− 13.68	− 0.21	< .001

M, Mean; SD, Standard Deviation; *t*-value from paired *t*-test; Cohen’s *d*, Effect size

**Fig. 4:**
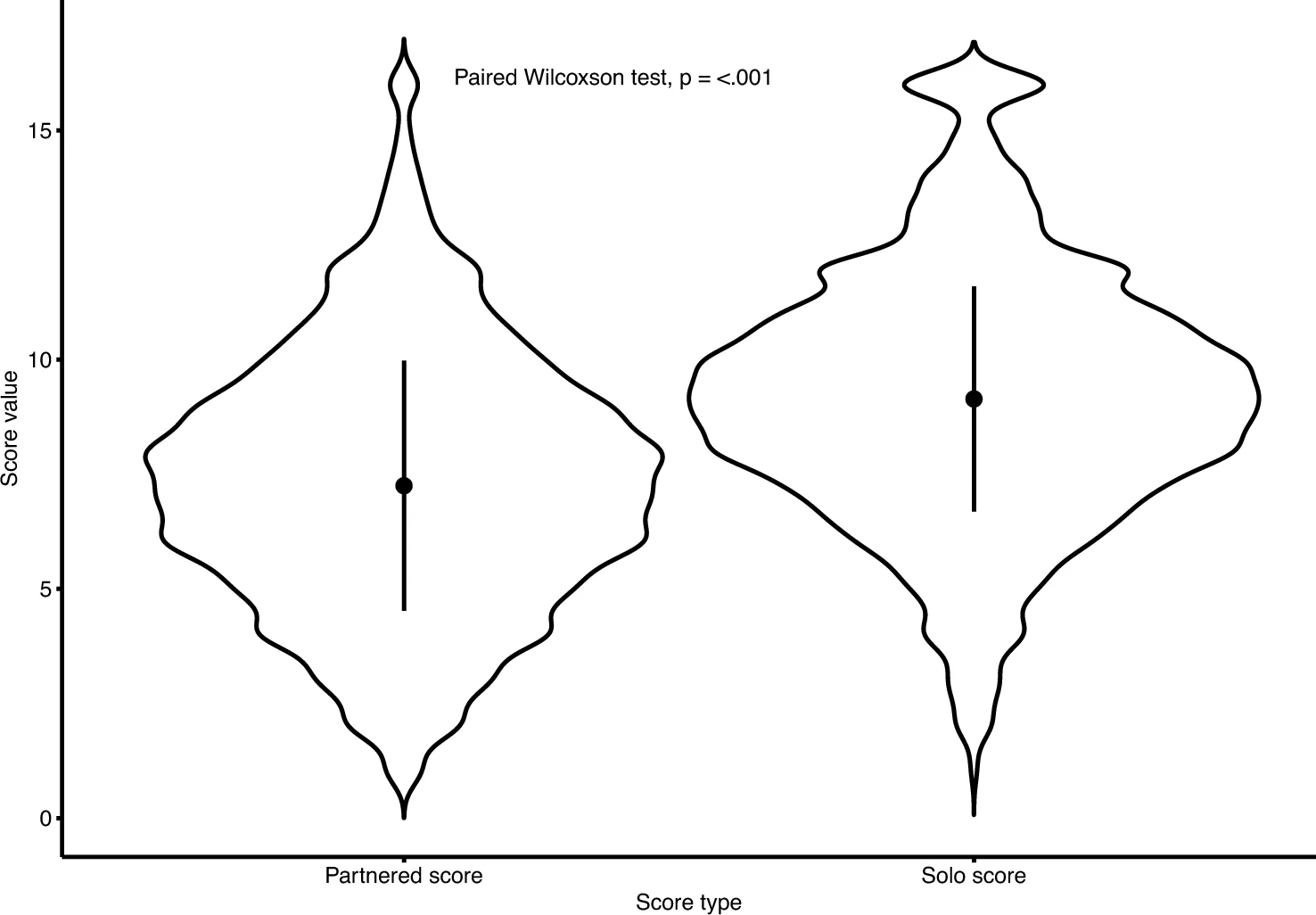
Overall orgasm frequency from solo versus partnered sexual activities

A Wilcoxon signed-rank test was conducted to compare overall orgasm frequency in solo versus partnered sexual activities, using weighted scores that accounted for both engagement frequency and orgasm frequency per activity. The results indicated a statistically significant difference between Solo Score (*Mdn* = 9.14, *Q*1 = 7.5, *Q*3 = 10.86) and Partner Score (*Mdn* = 7.25, *Q*1 = 5.40, *Q*3 = 9.00), *W* = 45,703,384, *p* < .001, *r* = 0.48. The distribution of scores, visualized in Fig. 4, shows that Solo Score was consistently higher than Partner Score, suggesting that individuals reported more frequent orgasms in solo sexual contexts than in partnered sexual contexts, even when accounting for how often each activity was engaged in.

## Discussion

In the current study, we sought to explore women’s orgasm frequency and sexual satisfaction among a wide variety of both partnered and solo sexual behaviors in a very large sample of women who use the Flo smartphone app. Compared with previous research, the current study assessed a larger variety of both partnered and solo sexual activities among women and their relationship to orgasm and satisfaction, using a relatively diverse sample with respect to sexual identities, age, and geographic location. We sought to describe women’s engagement in these varied solo and partnered sexual activities and then assess the associations between orgasms and sexual satisfaction.

### Are Orgasms Associated with Sexual Satisfaction?

The present study found that overall orgasm frequency was positively associated with overall sexual satisfaction, which is consistent with previous research (Dienberg et al., 2023; Frederick et al., 2017; Kontula & Miettinen, 2016; Leonhardt et al., 2018, 2024; Macedo et al., 2023; Pascoal et al., 2018). However, the strength of this association varied by the context of the orgasms. Despite the finding that women reached orgasm significantly more frequently during solo sexual activities than partnered sexual activities, even when the activities were nearly identical between contexts, greater orgasm frequency during *partnered* sexual activity was a stronger positive predictor of sexual satisfaction than solo orgasm frequency. That is, although not as reliable of a producer of orgasms as solo sexual activities, partnered orgasms have a stronger association with sexual satisfaction than solo orgasms. This finding potentially points to another orgasm gap, the gap between solo and partnered sexual activities.

These findings also give us greater insight into the complexity of the relationship between orgasm frequency, sexual variety, and sexual satisfaction. Sexual satisfaction is not exclusively about orgasm frequency. Instead, orgasms in a partnered context are more strongly associated with sexual satisfaction than orgasms in a solo context. There may be several potential reasons for this.

First, for women, partnered sexual activities may not carry the same societal stigma or shame as solo sexual activities. Women have repeatedly reported negative emotions during self-stimulation, including shame and guilt, that do not manifest in partnered sexual experiences (Cervilla & Sierra, 2022; Foust et al., 2022; Soares et al., 2024). Second, it is possible that due to the orgasm gap between solo and partnered sexual activities, orgasms in the context of partnered sexual experiences may be experienced as more impactful or meaningful (Hoy et al., 2022). Third, unlike solo sexual activities, partnered sexual activities are relational and include not only emotional connection but also connection through communication and shared pleasure (Goldey et al., 2016; Mangas et al., 2025; McClelland, 2014). Indeed, women in this study who orgasm more frequently during partnered sex reported that emotional, relational, and partner-based factors play a key role in their orgasm occurrence/experience. Many different variables contribute to overall sexual satisfaction, and the present study explored only a few potential variables that had not been examined with such nuance before.

### Is Sexual Variety Associated with Sexual Satisfaction and Orgasm?

Engaging in a wider variety of sexual activities was a positive predictor of orgasm frequency and sexual satisfaction in partnered sexual activities but not in solo sexual activities. The importance of sexual variety in partnered sexual contexts has been reported in several previous studies (Frederick et al., 2017; Townes et al., 2020; Willis et al., 2018). It may be that limited partnered sexual scripts that focus on the “phallocentric imperative” and thus lack variety account to some degree for this finding (Willis et al., 2018). Essentially, in heterosexual relationships, increased sexual variety beyond penile-vaginal penetration allows for a greater possibility of orgasm and increased sexual satisfaction. Additionally, couples who incorporate greater variety in their sexual activities need to have stronger sexual communication skills (Frederick et al., 2017), which is possibly an indicator of increased sexual vulnerability, sexual trust, and sexual connection. Given the mutuality of these sexual activities, engaging in a larger variety may increase mutual pleasure. Along these lines, sexual variety may be a manifestation of the partners’ interest in each other, playfulness, curiosity, confidence, and comfort.

In contrast to partnered sexual activities, a wider variety of solo sexual activities was negatively associated with sexual satisfaction. Additionally, engaging in a wider variety of solo sexual activities had no relationship to orgasm frequency.

### Engagement in Sexual Activities

Women reached orgasm significantly more frequently during solo sexual activities compared with partnered sexual activities (Blair et al., 2018; McElroy & Perry, 2024), even when the activities were nearly identical between contexts. This pattern was particularly strong for sexual activities that involved the use of sexual accessories (e.g., vibrators or erotica). This robust finding that women reach orgasm more consistently with solo sex compared with partnered sex is not surprising, given that solo sex allows for greater control of type and intensity of stimulation, stimulation is not dependent on partner skill, desire to engage in solo sex is not dependent on partner interest, and there is no pressure to perform for or please a partner, which may allow for the greater ability to be present in one’s body and attend to internal cues of desire and arousal (Dixon et al., 2024; Wiederman, 2000). Women have a “home court” advantage in knowing themselves and being in control. Consistent with this, women in this study reported that solo orgasms were more influenced by autonomy, lack of performance pressure, and greater control over stimulation as compared to partnered orgasms.

What we did not find is a direct connection between solo orgasm frequency and overall sexual satisfaction. In light of the literature on masturbation as a strategy to compensate for lack of access to partnered sex or insufficiently frequent or insufficiently satisfying partnered sex, higher rates of masturbation, and by extension more frequent orgasms from masturbation, may reflect lower levels of sexual satisfaction (Cervilla et al., 2024). Perhaps it is the relational aspect of orgasm, in relation to a partner, that makes orgasm a sexually satisfying experience versus a purely physiologically gratifying experience. This begs the question of how women define sexual satisfaction. Relationship satisfaction is a robust predictor of sexual satisfaction (Rausch & Rettenberger, 2021), which suggests that sexual satisfaction is heavily influenced by relational aspects.

In the present study, clitoral stimulation was the most frequently engaged in solo sexual activity. Surprisingly, it was also among the most frequently engaged in *partnered* sexual activity, contrasting with previous research that has reported vaginal penetration as the most frequently engaged in partnered behavior (Herbenick et al., 2010; Richters et al., 2006). While vaginal penetration only was the partnered activity most consistently engaged in every time participants had partnered sex, it was not the most frequently engaged in partnered activity overall. Instead, clitoral stimulation by a partner was reported by the highest percentage of respondents as occurring at least sometimes, even if not every time. Vaginal penetration with clitoral stimulation had the highest mean frequency score and was the second most common sexual activity reported as occurring every time.

This distinction between widespread engagement across participants (most frequently engaged in activities) and consistency within individuals (most consistently engaged in every time) highlights the importance of differentiating between how common an activity is among individuals overall versus how reliably it occurs within individual sexual encounters. Although our study did identify that vaginal penetration only was the most common sexual activity engaged in “every time” during partnered sex, it was ultimately not the most frequent partnered sexual activity. Thus, these findings likely reflect the finer granularity with which sexual engagement, frequency, and variety were assessed in the present study, compared with prior research (e.g., Herbenick et al., 2010). However, it is possible that the way the question was phrased contributed to the difference in our findings compared with others, as we specifically asked how often participants engaged in specific sexual activities in the timeframe of the past six months, rather than asking about their last sexual encounter.

Another possible explanation for this shift from vaginal penetration to increased clitoral stimulation may be a result of sample composition: just over one third (38%) of our sample was between the ages of 18 and 25, thereby including the experiences of Gen Z alongside those of previous generations. Although Herbenick et al. (2010) used a national probability sample with participants ranging in age from 14 to 94, the youngest members of their sample were from the Millennial generation. Given increased access to sexual health information online (Chen & Wang, 2021; Gottfried, 2024), social media’s focus on body positivity (Cohen et al., 2019, 2021), and the societal shift towards focusing on sexual pleasure as a fundamental right (Declaration on Sexual Pleasure|The World Association For Sexual Health [WAS], 2019; Ford et al., 2019), younger cohorts may be more likely to prioritize clitoral stimulation during partnered sex. Future research should further explore generational differences in sexual practices to better assess how sexual scripts are changing and the association between such changes and sexual satisfaction outcomes.

### Clinical Implications

There are several clinical implications of these findings. First, our finding that orgasm frequency from partnered, but not from solo sexual experiences, is a predictor of sexual satisfaction, highlights the value placed on partnered sex. Exploring emotional and relational aspects as key components of sexual pleasure and helping clients engage in sexual communication to enhance shared pleasure may assist in improving overall sexual satisfaction. Clinicians need to work with clients to help them shift from a performance-based, orgasm-oriented model of sexual satisfaction to a model of sexual pleasure and connection, similar to the Good Enough Sex Model, which emphasizes pleasure over performance and highlights the importance of sexuality as relational (McCarthy et al., 2024).

Second, there is an orgasm gap between solo and partnered sexual activities. Learning how to comfortably give and receive sexual feedback, explore sexual interests with a partner, and essentially bring the non-pressured solo sexual ingredients into a partnered sexual context may bridge this gap. Sex education that teaches the importance of and techniques to implement sexual communication could mediate this discrepancy (Mallory, 2022). When this gap is identified in sex therapy, sexual communication skills training may be a useful treatment intervention.

Third, these findings normalize a wide variety of solo and partnered sexual activities. Regarding solo activities, these findings may help alleviate the stigma and shame around solo sexual activities. Regarding partnered sexual activities, these findings provide more realistic sexual expectations. For example, despite vaginal penetration only being reported as the activity most engaged in “every time” by participants during partnered sex, women report that vaginal penetration is not the most common nor most pleasurable sexual activity. In other words, what is commonly shown in movies and media about the typical heteronormative sexual script that involves penile-vaginal penetration, culminating in the male partner’s orgasm, is indeed a common behavior, but not one that brings women the most pleasure.

### Strengths and Limitations

To our knowledge, the current study is the most comprehensive survey to date of women of all adult ages, assessing a much broader range of partnered and solo sexual behaviors than others before. The broad range of sexual activities assessed, both partnered and solo, is one major strength of this study. Few previous studies have looked at a wide range of sexual activities (e.g., Frederick et al., 2017), especially in both solo and partnered contexts. Another major strength is that this was a very large sample of high-quality data due to our recruitment method through the Flo app. Bots and synthetic users (i.e., those run by artificial intelligence) are likely not using smartphone apps for health tracking, which makes recruitment through these health apps a significant advantage for avoiding fabricated data.

There are several limitations of the present study. While the demographic spread of the respondents is broad, the majority of women were located in the US (36%), heterosexual (71%), White, European American, or Caucasian (56%), and held a Bachelor’s level degree or higher (52%). As age data was obtained from participants’ app data, relying on self-reported data entered to create a user account may have introduced inaccuracies in the data set. This means that our findings primarily reflect the experiences of younger, heterosexual, cisgender women who are digitally literate and use a menstrual cycle tracker app. This limits the generalizability of our findings, as they may not apply to older, less educated, more ethnically/racially diverse, or gender-diverse populations, as well as those lacking the digital skills or access to use a cycle tracking app or those who select not to use one.

Flo app users may be more open to and comfortable with reporting on their sexuality and sexual behaviors than women among the general public. They may also be more sexually health-literate, as app users have access to a range of menstrual cycle and sexual education content, as well as the ability to log sex events and a wide range of menstrual health-related symptoms. It is also possible that women who use cycle-tracking apps are more sexually active than women in the general public. However, reasons for using cycle tracking apps are varied and individual, and include a greater understanding of menstrual cycle symptoms and changes, knowledge of period start dates, insights into one’s body, and facilitating informed conversations with healthcare professionals (Broad et al., 2022; Levy & Romo-Avilés, 2019).

Another limitation was that the survey was only available in English, limiting the reach among those who use the app in other languages. This likely led to fewer respondents in countries and world regions where English is not a dominant language. Future work would benefit from extending this research into other languages to gain a deeper understanding of women’s experiences in varying locations. In addition, as with all self-reported surveys, there is a potential for recall bias, leading to over- or underreporting of frequency of sexual activity, satisfaction, and orgasm. While we tried to assess sexual satisfaction as a holistic measure, our approach to survey participants about their satisfaction with their overall sex life does not provide specific insights into how respondents conceptualize their satisfaction. Indeed, it is possible that most participants viewed sexual satisfaction specifically in the context of a partnered sexual relationship.

Further, the cross-sectional nature of this study prevents causal interpretations. To properly assess the association between variety, orgasm frequency, and sexual satisfaction, longitudinal data would be needed to run a mediation analysis. It is feasible in the present study that women who are highly satisfied with their sex lives actively seek more variety in their sexual behaviors and that they also experience more frequent orgasms. Finally, while this work is the largest and most comprehensive study of its type, the large sample size does allow for statistically significant differences to be detected whose effect sizes are not meaningful at a clinical or practical level. We have included relevant indicators in the tables for transparency. Areas where we have highlighted small-magnitude effects should be further explored in more targeted and detailed studies.

### Future Directions and Conclusion

The current study reported frequencies of specific solo and partnered sexual activities, orgasm frequency, and sexual satisfaction, as well as associations between these factors among a large sample of Flo app users. The present study found that greater orgasm frequency was associated with greater sexual satisfaction for partnered, not solo, sexual activities. Additionally, a wider variety of sexual activities was positively associated with orgasm frequency and sexual satisfaction for partnered, not solo, sexual activities. Overall, these findings suggest that sexual satisfaction is *not* all about the orgasm. Rather, orgasm frequency and sexual variety during partnered sexual activity serve as key contributors to sexual satisfaction. However, orgasm frequency and variety during solo sexual activity do not predict sexual satisfaction, and in some cases predict lower sexual satisfaction. We suspect that this finding is due to participants’ general sexual satisfaction being driven largely by partnered sexual experiences rather than solo sexual experiences.

These findings raise several questions. First, if the emotional and relational aspect of partnered sex is a significant driver for sexual satisfaction, is there any way to capture that interpersonal aspect during solo sexual activities? What role might artificial intelligence have in enhancing solo sexual experiences (Belk, 2022)? Second, when emotional and relational factors are fulfilled, how important is the orgasm gap? That is, if attending to emotional and relational factors is more sexually satisfying for women than orgasmic frequency, should the focus of future research be on exploring the emotional/relational gap (if one exists) versus on sexual performance (i.e., orgasm frequency)? Future studies that track not only changes in sexual variety, orgasm frequency, and sexual satisfaction throughout a developing relationship but also assess emotional/relational factors, such as sexual communication, emotional closeness, relationship satisfaction, trust, and commitment, would help clarify these questions. Finally, we hope that the current study provides clinicians working in this field with a deeper understanding of the breadth of the sexual experiences of women and how this relates to orgasms and their sexual satisfaction.

## Supplementary Information

Below is the link to the electronic supplementary material.

Supplementary file1 (DOCX 124 KB)

## Data Availability

The deidentified data that support the findings of this study are available from Flo Health UK Limited, but restrictions apply to the availability of these data, so they are not publicly available. Data are, however available from the authors upon reasonable request and with the permission of Flo Health UK Limited.

## Code Availability

Data was collated using R version 4.4.0. The code used to analyze the dataset is available upon reasonable request from the corresponding author and with the permission of Flo Health UK Limited.
