# Supplementary material for: Sexual Satisfaction and Orgasm Experiences During Partnered and Solo Sex Among 27,931 Users of the Flo App
Source: Arch Sex Behav. 2026 Jun 9;55(6):2483–503. doi: 10.1007/s10508-025-03393-y (PMC13597633; doi:10.1007/s10508-025-03393-y)
Supplement: Supplementary file 1 [file 10508_2025_3393_MOESM1_ESM.docx]

**Appendix**

**Figure A1**

**Flowchart of responses through the study**

**Table A1**

**Satisfaction and orgasm frequency scores from responses to the satisfaction with overall sex life and orgasm frequency questions**

|  | **Overall (N=27931)** |
| --- | --- |
| **Satisfaction score** |  |
| N-Miss | 1826 |
| Mean (SD) | 3.597 (1.225) |
| Range | 1.000 - 5.000 |
| **Orgasm frequency score** |  |
| N-Miss | 1553 |
| Mean (SD) | 3.645 (1.445) |
| Range | 0.000 - 5.000 |

**Note.** SD = Standard Deviation.

**Table A2**

**Pearson correlation matrix for supplementary material**

|  | **Satisfaction** | **Overall Orgasm Frequency** | **Partnered score** | **Solo score** | **Partnered count** | **Solo count** | **Partnered orgasm frequency** | **Solo orgasm frequency** |
| --- | --- | --- | --- | --- | --- | --- | --- | --- |
| **Satisfaction** | 1 |  |  |  |  |  |  |  |
| **Overall Orgasm Frequency** | 0.376*** | 1 |  |  |  |  |  |  |
| **Partnered score** | 0.350*** | 0.498*** | 1 |  |  |  |  |  |
| **Solo score** | 0.029*** | 0.052*** | 0.176*** | 1 |  |  |  |  |
| **Partnered count** | 0.271*** | 0.260*** | 0.186*** | -0.011 | 1 |  |  |  |
| **Solo count** | 0 | 0.068*** | -0.006 | -0.093*** | 0.344*** | 1 |  |  |
| **Partnered orgasm frequency** | 0.252*** | 0.330*** | 0.264*** | -0.018** | 0.191*** | 0.077*** | 1 |  |
| **Solo orgasm frequency** | -0.066*** | -0.071*** | -0.068*** | 0.105*** | -0.005 | 0.072*** | 0.452*** | 1 |
| **Note.** **** p<.001; ** p<.01; * p<.05* | | | | | | | | |
